# Multiparity increases the risk of diabetes by impairing the proliferative capacity of pancreatic β cells

**DOI:** 10.1038/s12276-023-01100-2

**Published:** 2023-10-31

**Authors:** Joon Ho Moon, Joonyub Lee, Kyun Hoo Kim, Hyun Jung Kim, Hyeongseok Kim, Hye-Na Cha, Jungsun Park, Hyeonkyu Lee, So-young Park, Hak Chul Jang, Hail Kim

**Affiliations:** 1 Graduate School of Medical Science and Engineering, Korea Advanced Institute of Science and Technology, Daejeon, 34141 Korea; 2 Department of Internal Medicine, Seoul National University Bundang Hospital, Seoul National University College of Medicine, Seoul, Korea; 3 Division of Endocrinology and Metabolism, Department of Internal Medicine, Seoul St. Mary’s Hospital, College of Medicine, The Catholic University of Korea, Seoul, Republic of Korea; 4 Biomedical Research Center, Korea Advanced Institute of Science and Technology, Daejeon, 34141 Korea; 5 Department of Biochemistry, College of Medicine, Chungnam National University, Daejeon, Korea; 6 Department of Physiology, College of Medicine, Yeongnam University, Daegu, Korea

**Keywords:** Gestational diabetes, Type 2 diabetes

## Abstract

Pregnancy imposes a substantial metabolic burden on women, but little is known about whether or how multiple pregnancies increase the risk of maternal postpartum diabetes. In this study, we assessed the metabolic impact of multiple pregnancies in humans and in a rodent model. Mice that underwent multiple pregnancies had increased adiposity, but their glucose tolerance was initially improved compared to those of age-matched virgin mice. Later, however, insulin resistance developed over time, but insulin secretory function and compensatory pancreatic β cell proliferation were impaired in multiparous mice. The β cells of multiparous mice exhibited aging features, including telomere shortening and increased expression of *Cdkn2a*. Single-cell RNA-seq analysis revealed that the β cells of multiparous mice exhibited upregulation of stress-related pathways and downregulation of cellular respiration- and oxidative phosphorylation-related pathways. In humans, women who delivered more than three times were more obese, and their plasma glucose concentrations were elevated compared to women who had delivered three or fewer times, as assessed at 2 months postpartum. The disposition index, which is a measure of the insulin secretory function of β cells, decreased when women with higher parity gained body weight after delivery. Taken together, our findings indicate that multiple pregnancies induce cellular stress and aging features in β cells, which impair their proliferative capacity to compensate for insulin resistance.

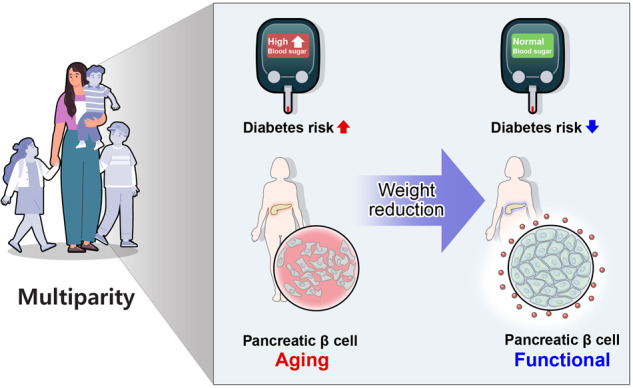

## Repeated pregnancies and their impact on maternal diabetes

Researchers used a mouse model to investigate the impact of multiple pregnancies on long-term metabolic health of female, discovering that pancreatic β cells in multiparous mice lost their proliferative capacity, leading to hyperglycemia. Transcriptomic analyses showed features of senescence and increased cellular stress in these cells. In humans, women with a higher number of pregnancies had reduced glucose tolerance compared to those with fewer deliveries. Multiparous women who gained weight after delivery had impaired insulin secretory function while multiparous women who maintained or lost weight maintained their pancreatic β cell function. The study highlights the need for careful monitoring and lifestyle modifications, such as weight reduction, in women who have undergone multiple pregnancies to reduce the risk of postpartum diabetes.

## Introduction

Pregnancy imposes a substantial metabolic burden on women through weight gain and insulin resistance. Women gain more than 10 kg in body weight during the gestation period^1^, while pregnancy-related hormones, including tumor necrosis factor α, placental lactogen, growth hormone, cortisol, and progesterone, increase insulin resistance during pregnancy^2^. Thus, women who undergo multiple pregnancies are likely to have an increased risk of diabetes in the postpartum period^3,4^.

During pregnancy, pancreatic β cell mass undergoes dynamic changes to compensate for increased metabolic demands. At mid-gestation, the β cell mass expands by up to 3–4-fold in rodents and 1.4-fold in humans^5,6^. At the end of gestation, the β cell mass regresses to the pregestational level^7^. Therefore, β cells undergo expansion and regression throughout gestation, which is expected to increase their cellular stress. Although counterregulatory mechanisms exist to compensate for the increased cellular stress during gestation^8–10^, β cells are likely to be in a state of distress after multiple pregnancies^11^.

Previous clinical studies have demonstrated an increased risk of postpartum diabetes associated with grand multiparity^12,13^, but a clear understanding of the metabolic changes that occur during and after pregnancy is lacking. In particular, mechanistic studies examining alterations in pancreatic β cells and other peripheral organs following multiple pregnancies are lacking. This unmet need has led to the hypothesis that the repeated expansion and regression of β cells by multiple pregnancies could potentially induce cellular distress that would impede their compensatory mechanisms to handle the insulin resistance that occurs with aging, thereby placing mothers at an increased risk of postpartum diabetes. To demonstrate this notion, we characterized the impact of multiple pregnancies on the metabolic health of postpartum female humans and mice. Our analysis in a mouse model revealed that β cells of multiparous mice lost their proliferative capacity and became unable to compensate for the increasing insulin demand, leading to hyperglycemia. Transcriptomic analyses of multiparous β cells showed features of senescence and increased cellular stress, which could underlie the impaired proliferative capacity of these cells. Human studies have shown that women with a higher number of pregnancies are intolerant to glucose compared to those who have delivered less and that postpartum weight reduction in multiparous women results in improved glucose tolerance, insulin sensitivity, and insulin secretory function. This study provides potential implications for women who have undergone multiple pregnancies to be carefully monitored for the future development of diabetes and to have lifestyle modifications, including weight reduction, be recommended.

## Materials and methods

### Animal experiments

**Fig. 1:**
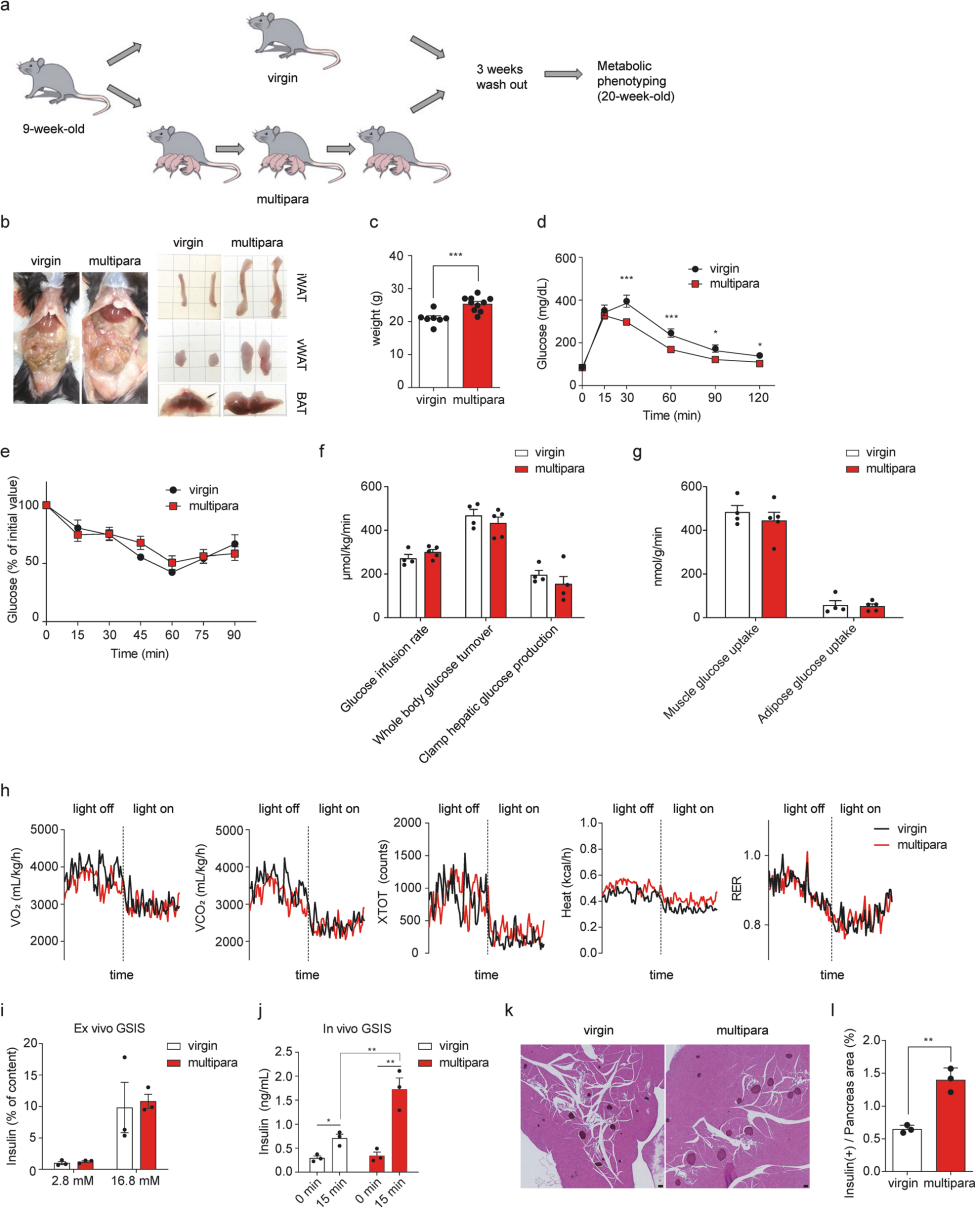
Multiparity improves glucose tolerance by increasing β cell mass. **a** Schematic depicting the mouse model for multiple pregnancy. Age-matched female C57BL/6 J mice were randomly assigned to the virgin or multipara groups and mated to give birth three times consecutively. Metabolic phenotypes were evaluated at 3 weeks after the last delivery. **b** Gross images of virgin and multiparous mice and their inguinal white adipose tissue (iWAT), visceral WAT (vWAT), and brown adipose tissue (BAT) dissected at 3 weeks after the last delivery. **c** Body weight and **d** intraperitoneal glucose tolerance test data obtained after an overnight fast (2 g/kg); *n* = 7 for virgin, *n* = 10 for multipara. **e** Intraperitoneal insulin tolerance test after a 6 h fast (0.75 U/kg; *n* = 3 per group). **f, g** Glucose infusion rate, whole body glucose turnover, clamp hepatic glucose production, and **g** glucose uptake from soleus muscle and retroperitoneal adipose tissue were measured by the hyperinsulinemic-euglycemic clamp method in virgin and multiparous mice (*n* = 4 for virgin and *n* = 5 for multipara). **h** Indirect calorimetric analysis of virgin and multiparous mice. Oxygen consumption (VO_2_), carbon dioxide consumption (VCO_2_), total activity at the x-axis (XTOT), heat production, and the respiratory exchange ratio (RER) were recorded (*n* = 4 per group). **i** Islets were incubated for 15 min with 2.8 or 16.8 mM glucose, and the secreted insulin concentrations were measured with ELISA (*n* = 3 per group). **j** Plasma insulin concentrations were measured after intraperitoneal glucose injection (2 g/kg; *n* = 3 per group). **k** Representative images of pancreatic islets of virgin and multiparous mice; insulin was immunohistochemically (brown) stained with hematoxylin and eosin counterstaining. Scale bar: 50 μm. **l** The β cell area was quantified as the percentage of the insulin-positive area relative to the area of the entire pancreas. **k, l** *n* = 3 per group. (Data are expressed as the mean ± SEM. *P < 0.05, **P < 0.01 and ***P < 0.001, as determined by Student’s *t* test (**c**–**g, l**) or one-way ANOVA with Tukey’s *post hoc* test (**i, j**).

**Fig. 2:**
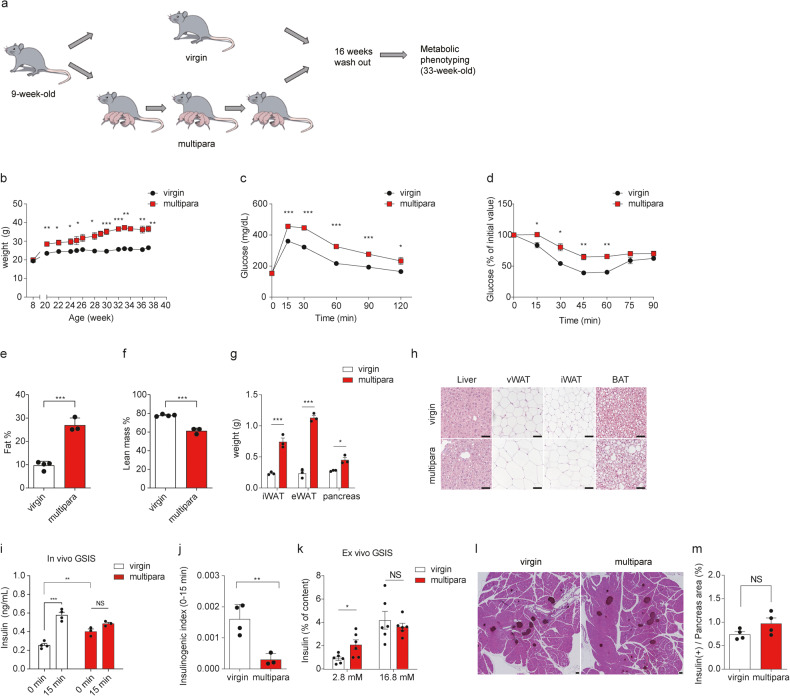
Multiparous β cells fail to compensate for insulin resistance. **a** Schematic depicting the mouse model for repeated pregnancy, evaluated at 16 weeks after the last delivery (34 weeks of age). **b** Body weight changes in virgin and multiparous mice. **c** Intraperitoneal glucose tolerance test after an overnight fast (2 g/kg). **d** Intraperitoneal insulin tolerance test after a 6 h fast (0.75 U/kg). **b–d** *n* = 4 per group). **e** Fat and **f** lean mass proportions in virgin and multiparous mice (*n* = 4 for virgin, *n* = 3 for multipara). **g** Weights of the indicated tissues (*n* = 3 per group). **h** Representative images of liver, vWAT, iWAT, and BAT (*n* = 3 per group, scale bar: 50 μm). **i** Plasma insulin concentrations and **j** the insulinogenic index were measured after intraperitoneal glucose injection (2 g/kg; *n* = 4 per group). **k** Islets were incubated for 15 min with 2.8 or 16.8 mM glucose, and the secreted insulin concentrations were measured with ELISA (*n* = 6 per group). **m** Representative images of pancreatic islets of virgin and multiparous mice; insulin was immunohistochemically detected (brown) with hematoxylin and eosin counterstaining. Scale bar: 50 μm. **l** The β cell area was quantified as the percentage of the insulin-positive area relative to the area of the entire pancreas. **l**, **m** *n* = 4 per group. Data are expressed as the mean ± SEM. NS not significant. *P < 0.05, as determined by Student’s *t* test (**b**–**g, j, m**) or one-way ANOVA with Tukey’s *post hoc* test (**i, k**).

For the multiparity model experiments, 9-week-old female littermate mice (C57BL/6 J, The Jackson Laboratory) were randomly assigned to the virgin or multipara groups. These mice were mated and gave birth three times consecutively (multipara), and their virgin littermates were used as controls (virgin). Metabolic phenotypes were evaluated at least 3 weeks after the last delivery (3-week washout for the Fig. 1 cohort and 16-week washout for the Fig. 2 cohort). For the S-961 studies, 100 nmol/kg mouse S-961 (insulin receptor antagonist, Novo Nordisk) was intraperitoneally administered twice a day for 3 or 7 days. All mouse studies were approved by the Institutional Animal Care and Use Committee (IACUC) of Korea Advanced Institute of Science and Technology. All experiments were performed in accordance with the relevant guidelines and regulations. Further details on the metabolic assessments are described in the Supplementary Methods.

### Islet studies

Pancreatic islets were isolated from C57BL/6 J virgin and multipara mice as described previously^14^. For ex vivo glucose-stimulated insulin secretion (GSIS), the retrieved islets were incubated for 15 min in 2.8 mM glucose (basal, low) or 16.8 mM (stimulated, high) glucose-containing buffer, and the secreted insulin level was measured. Insulin secretion was normalized to the content of insulin extracted from the islets using acid-ethanol. Further details are described in the Supplementary Methods.

### Quantitative reverse transcription-PCR (qRT‒PCR) and RNA sequencing

RNA was extracted from pancreatic islets using TRIzol (Invitrogen, 15596026). *Actb* was used as an internal control. Telomere length was measured using qRT‒PCR, as described in detail elsewhere^15^. The primers used to evaluate the expression of each gene are listed in Supplementary Table 2.

For RNA sequencing, samples with an RNA integrity number (RIN) > 8.0 were selected, and 1 μg of total RNA was applied to construct cDNA libraries using an Illumina TruSeq Stranded mRNA kit (RS-122-9004DOC). Further details are described in the Supplementary Methods.

### Single-cell RNA sequencing

Isolated pancreatic islets from C57BL/6 J mice were dissociated into single cells with gentle pipetting in the presence of 0.25% trypsin for 5 min. Dissociated cells were subjected to the 10X Genomics Chromium system, and cDNA libraries were constructed using Chromium Single-Cell 3′ Reagent Kits (v3.1 Chemistry). The libraries were sequenced using a HiSeq X Ten (Illumina). The sequenced data were aligned and analyzed using Cell Ranger (3.1.0) and Seurat v3.0. Cells that met the criteria of nGene_RNA > 200, nFeature_RNA < 6000, nCount_RNA < 60,000, and percent.mt <10 were used for the analysis. Gene set enrichment analysis (GSEA) was performed with fgsea (v.1.12.0)^16^. GSEA was based on the Molecular Signatures Database (msigdb c5.all.v7.1.symbols, https://data.broadinstitute.org/gsea-msigdb/msigdb/release/7.1/). The number of permutations adopted was 1000, and *p* < 0.05 was regarded as statistically significant. Further details are described in the Supplementary Methods.

### Immunostaining

Immunofluorescence and immunohistochemical staining were performed using formalin-fixed paraffin-embedded mouse tissues following a standard protocol. Details including the measurement of β cell mass and proliferation are described in the Supplementary Methods.

### Human studies

For human studies, we conducted a multicenter prospective cohort study among subjects with a first-time diagnosis of gestational diabetes mellitus (GDM) or gestational impaired glucose tolerance (GIGT)^17^. Subjects were recruited from August 1995 to May 1997 from four centers in Korea. Further details on human studies, including diagnostic criteria for GDM and GIGT, are described in the Supplementary Methods.

Women with GDM or GIGT who visited the initial postpartum evaluation were enrolled in this study. The initial postpartum follow-up visit was performed at 2 months postpartum, and annual follow-up visits were made thereafter. Women who had one to three pregnancies were categorized as ‘parity-low’, and those with four or more were categorized as ‘parity-high’. A total of 455 women were included in the analysis (parity-low, *n* = 376; parity-high, *n* = 79). The median duration of follow-up was 4.0 (interquartile range, 2.2–5.0) years after delivery.

A standard 75 g OGTT and anthropometric measures were measured at each visit. Insulin sensitivity was assessed by the Matsuda index as follows: 10,000/√[(fasting glucose) × (fasting insulin) × (mean glucose) × (mean insulin)]. The insulinogenic index was used to estimate insulin secretion as follows: (insulin [30 min] – insulin [0 min])/(glucose [30 min] – glucose [0 min]). The disposition index was used to evaluate the composite of insulin secretion considering the degree of insulin sensitivity as follows: (Matsuda index) × (insulinogenic index).

All subjects participated voluntarily, and informed consent was obtained from each subject. This study was approved by the Institutional Review Board of Seoul National University Bundang Hospital (IRB Number: B-1903-526-107) and was conducted according to the requirements of the Declaration of Helsinki.

### Statistical analysis

All data are presented as the mean ± standard error of measurements (SEM) for continuous variables or as a number (percentage) for nominal variables. The statistical significance was measured by Student’s *t* test (two-tailed) or ANOVA (with Tukey’s *post hoc* test) for continuous variables and the χ^2^ test for categorical variables. Statistical analyses were conducted using SPSS version 22 (IBM). The level of statistical significance is given as *P < 0.05, **P < 0.01 and ***P < 0.001.

## Results

### Multiparity improves glucose tolerance by increasing β cell mass

To investigate whether multiparity increases the risk of diabetes after delivery, we established a mouse model of multiparity (Fig. 1a). Nine-week-old female C57BL/6 J mice were mated to give birth three times consecutively (multipara), and their metabolic characteristics were analyzed at 3 weeks after the last delivery. Their virgin littermates were used as controls (virgin). Multiparous mice showed increased adiposity in their inguinal white adipose tissue (iWAT) and visceral white adipose tissue (vWAT) compared to that of virgin mice (Fig. 1b). The body weight of multiparous mice was higher than that of virgin mice (Fig. 1c). However, multiparous mice exhibited improved glucose tolerance, and their insulin tolerance was comparable to that of virgin mice (Fig. 1d, e). Insulin sensitivity was further assessed by hyperinsulinemic clamp study (Fig. 1f, g). The glucose infusion rate, glucose turnover, hepatic glucose production, and glucose uptake in the peripheral tissues of multiparous mice were comparable to those in virgin mice. In addition, energy expenditure and heat generation were not altered in multiparous mice (Fig. 1h). These data suggest that multiparity did not induce insulin resistance despite the increased adiposity but rather improved glucose tolerance.

To examine whether changes in the β cells of multiparous mice could account for the improvement in glucose tolerance in the absence of a change in insulin sensitivity, we assessed the β cell function of multiparous mice. An ex vivo glucose-stimulated insulin secretion (GSIS) assay performed using isolated islets showed that insulin secretion was comparable between virgin and multiparous mice (Fig. 1i). However, in vivo GSIS was potentiated in multiparous mice, although the basal insulin levels were comparable (Fig. 1j). Histological analysis revealed that the β cell mass was increased by 2.2-fold in multiparous mice (0.6% in virgin control mice and 1.3% in multiparous mice) (Fig. 1k, l). Taken together, these results show that although multiparous mice were more obese than virgin mice, they did not develop insulin resistance. Instead, glucose tolerance was improved, and the β cell mass remained increased in multiparous mice at 3 weeks after the last delivery.

### Multiparous mice develop insulin resistance without β cell compensation

Our finding that multiparous mice had improved glucose tolerance at 3 weeks after the last delivery contrasted with reported observations in humans^12,18^. We speculated that the phenotypes seen herein for multiparous mice could reflect the acute effects of multiparity, since the analyses in humans were performed at time points further out after the last delivery^19–22^. To avoid the acute effects of pregnancy and explore the long-term metabolic effects of multiparity, we assessed the phenotypes of multiparous mice at a longer period after the last delivery and compared them with age-matched virgin mice (Fig. 2a). Multiparous mice continuously gained weight for 16 weeks after the last delivery, reaching ~40 g at 34 weeks of age, and became glucose intolerant and insulin resistant (Fig. 2b–d). The fat mass was increased and the lean mass was decreased in multiparous mice, which reflected increases in the iWAT and vWAT masses (Fig. 2e–g). Hepatic steatosis was aggravated, the white adipocyte size was increased, and brown adipocytes were more whitened in multiparous mice than in virgin mice (Fig. 2h). These data suggest that multiparous mice are more prone to gaining weight and becoming insulin resistant over time than virgin mice.

Glucose intolerance develops when β cells fail to compensate for increased insulin resistance. In multiparous mice, we noted that insulin resistance developed and the glycemic profile became aggravated over time, even though the β cell mass of these mice at 3 weeks after the last delivery was larger than that of virgin mice. This prompted us to study β cell function in multiparous mice at 34 weeks of age to test whether multiparous β cells had properly compensated for insulin resistance. The fasting plasma insulin concentration was elevated and GSIS was blunted in multiparous mice, and these changes were reflected in a substantially decreased insulinogenic index (Fig. 2i, j). Likewise, ex vivo GSIS assays showed that basal insulin secretion was elevated under low-glucose conditions and that GSIS was impaired in islets from multiparous mice (Fig. 2k). Given that insulin secretion was not impaired in β cells of multiparous mice at 3 weeks after the last delivery, these data indicate that the insulin secretory function of β cells from multiparous mice deteriorated over time after the last delivery, along with the progression of insulin resistance.

To examine whether multiparous mice could further increase their mass in response to the development of insulin resistance, we measured the β cell mass in multiparous mice at 33 weeks of age. In multiparous mice, the β cell mass was increased by 2.2-fold at 3 weeks after the last delivery, before insulin resistance had developed (0.6% in virgin control mice and 1.3% in multiparous mice at 21 weeks of age) (Fig. 1l). However, at 34 weeks of age, there was no significant difference in the β cell mass between multiparous and virgin mice (0.7% and 1.0%, respectively) (Fig. 2l, m). Interestingly, insulin resistance and weight gain did not further increase the β cell mass in multiparous mice (1.3% at 21 weeks of age and 1.0% at 34 weeks of age) (Figs. 1l, 2m).

Taken together, these findings indicate that obesity and subsequent insulin resistance developed over time in multiparous mice, but these mice were not able to increase their β cell mass to compensate for the insulin resistance. In addition, β cell function declined over time in multiparous mice. Therefore, the glucose intolerance seen in multiparous mice could be attributed to insufficient β cell compensation for insulin resistance.

### Multiparity impairs the proliferative capacity of β cells

**Fig. 3:**
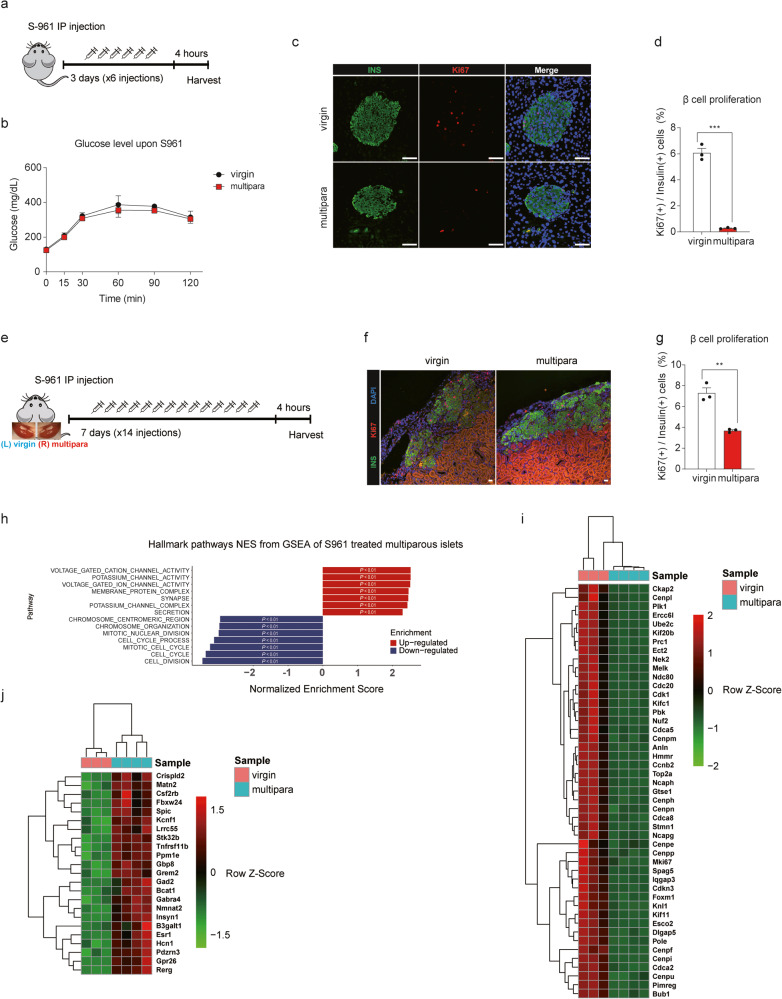
Multiparity impairs the proliferative capacity of β cells. **a** Schematic depicting S-961 (insulin receptor antagonist) administration to test the replicative capacity of β cells in virgin and multiparous mice. **b** Glucose concentrations after S-961 administration in virgin and multiparous mice. **c** Representative images of Ki67 (red) and insulin (green) immunofluorescence in pancreas sections after S-961 administration (scale bar: 50 μm). **d** The β cell proliferation rate was calculated as the percentage of insulin and Ki-67 copositive cells relative to all insulin-positive cells. **b**–**d** *n* = 3 mice per group. (e-g) Size-matched islets from virgin and multiparous mice were transplanted into the left and right renal capsules, respectively, of C57BL/6 J mice, which were then injected intraperitoneally with S-961. **e** Schematic depicting the administration of S-961 to mice transplanted with islets from virgin and multiparous mice into the left and right renal capsules, respectively. **f** Representative images of Ki67 (red) and insulin (green) immunofluorescence in kidney sections after S-961 administration (scale bar: 100 μm). **g** The β cell proliferation rate as the percentage of insulin and Ki-67 copositive cells relative to all insulin-positive cells. **f, g** *n* = 3 per group. (h-j) RNA sequencing of islets obtained from virgin (*n* = 3) and multiparous (*n* = 4) mice subjected to intraperitoneal injection of S-961. **h** Gene set enrichment analysis of virgin and multiparous islets. **i** Downregulated and **j** upregulated genes of multiparous islets compared to virgin islets. **i, j** Z Scores of DESeq2 normalized counts are annotated by the color scale. (Data are expressed as the mean ± SEM. *P < 0.05, **P < 0.01 and ***P < 0.001, as determined by Student’s *t* test).

The failure of multiparous mice to increase their β cell mass to compensate for insulin resistance suggested that multiple pregnancies per se may alter the characteristics of β cells prior to the development of insulin resistance. In this regard, we treated multiparous mice at 3 weeks after the last delivery with S-961, an insulin receptor antagonist that induces insulin resistance and β cell proliferation, and evaluated β cell proliferation in these mice (Fig. 3a). S-961 administration for 3 days induced glucose intolerance similarly in both virgin and multiparous mice (Fig. 3b). However, whereas S-961 induced robust β cell proliferation in virgin mice, this effect was not observed in multiparous mice (6.1% and 0.3%, respectively) (Fig. 3c, d). These data suggest that there may be a defect in the ability of β cells from multiparous mice to proliferate in response to insulin resistance.

To exclude the possibility that systemic changes in multiparous mice might inhibit β cell proliferation, we transplanted islets isolated from virgin and multiparous mice into the left and right kidneys of virgin mice, respectively, and treated the mice with S-961 for 7 days (Fig. 3e). In accordance with the above-described results, S-961 treatment robustly induced proliferation among the β cells from virgin mice in the left kidney but not in the β cells from multiparous mice in the right kidney (7.3% and 3.7%, respectively) (Fig. 3f, g). Taken together, these data indicate that the β cells of mice that underwent multiple pregnancies have a limited proliferative capacity even before the development of insulin resistance.

To gain insight into the molecular signature related to the impaired proliferative capacity of β cells from multiparous mice, we performed bulk RNA sequencing (RNA-seq) with islets isolated from virgin and multiparous mice treated with or without S-961 for 3 days (Figs. 1a, 3a). Analysis of bulk RNA-seq data obtained from the islets of virgin and multiparous mice without S-961 treatment identified a number of differentially expressed genes (DEGs); however, principal component analysis (PCA) did not show distinct changes in the expression of genes that would be expected to impair the proliferative capacity (Supplementary Fig. 1a, b). Most of the genes upregulated in islets from multiparous mice were previously reported to be upregulated during pregnancy (e.g., *Lrrc55, Matn2*, and *Lars2*)^9^. In contrast, bulk RNA-seq analysis of islets isolated from S-961-treated multiparous and virgin mice revealed distinct expression patterns upon PCA (Supplementary Fig. 1c, d). Gene set enrichment analysis (GSEA) further revealed that the expression levels of genes involved in pathways related to cell cycle regulation were downregulated in islets from S-961-treated multiparous mice, whereas those of genes involved in pathways related to voltage-gated cation channel, potassium channel activity, and membrane protein complex were upregulated (Fig. 3h). Regarding cell cycle-related genes, our analysis revealed that genes related to centromere proteins (*Cenpi* and *Cenpf*), cell cycle progression (*Cdk1, Cdk2*, and *Cdkn2d*) and other cell cycle regulations (*Hmmr, Stmn1*, and *Cdc20*) were downregulated in islets from S-961-treated multiparous mice (Fig. 3i). In contrast, relatively few genes were upregulated in islets from S-961-treated multiparous mice, and these included genes that were previously reported to be upregulated during pregnancy (e.g., *Matn2* and *Lrrc55*) (Fig. 3j). These transcriptomic changes suggest that β cells of multiparous mice are unable to activate the proliferation machinery in response to the increased metabolic demand.

### Increased cellular stress with senescence is seen in β cells of multiparous mice

**Fig. 4:**
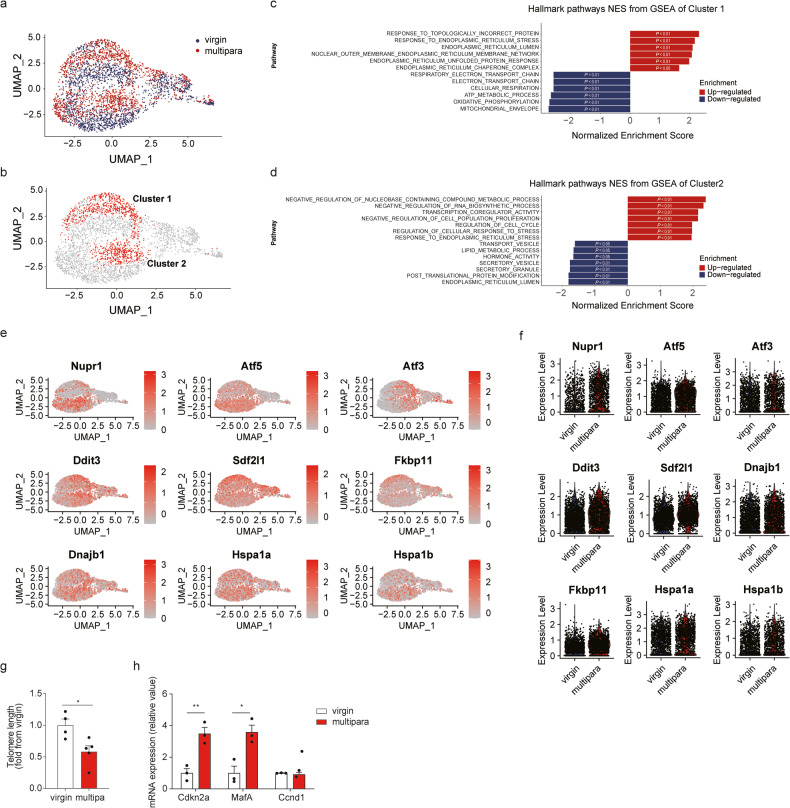
Multiparity increases cellular stress and accelerates the aging process of β cells. **a–f** Single-cell RNA sequencing of islets from virgin and multiparous mice (*n* ≥ 2 per group). **a, b** Multiparous mouse-specific β cell transcript clusters were annotated as Cluster 1 and Cluster 2 (Supplementary Fig. 2). **c, d** Pathways of genes enriched in **c** Cluster 1 and **d** Cluster 2 were analyzed using gene set enrichment analysis. **e, f** Stress-related genes visualized using UMAP (**e**) and a violin plot (**f**). **g** Telomere length was measured in islets from virgin (*n* = 4) and multiparous (*n* = 5) mice. **h** mRNA expression levels in islets from virgin and multiparous mice were assessed by qRT‒PCR (*n* = 3 per group). (Data are expressed as the mean ± SEM. *P < 0.05, **P < 0.01 and ***P < 0.001, as determined by Student’s *t* test).

The proliferative capacity of β cells was reduced in multiparous mice, but bulk RNA-seq of islets did not reveal the related genetic signature. A pancreatic islet is made up of various types of cells, and β cells themselves represent a heterogeneous population, only a small portion of which are proliferating at a given moment; thus, some transcriptomic information regarding the proliferative capacity of β cells could be masked in the bulk RNA-seq analysis performed on islets. To overcome this limitation, we performed single-cell RNA sequencing (scRNA-seq) with islet cells isolated from multiparous mice at 3 weeks after the last delivery and age-matched virgin mice. Islet cells from multiparous and virgin mice exhibited similar cellular proportions and similar expression patterns, except for those of β cells (Supplementary Fig. 2a, b, c). Using uniform manifold approximation and projection (UMAP), β cells were divided into seven subpopulations (Fig. 4a, Supplementary Fig. 2d). Of them, two distinct β cell subpopulations, Clusters 1 and 2, were present predominantly in β cells from multiparous mice and clusters 5 and 7 were present predominantly in β cells from virgin mice (Fig. 4b, Supplementary Fig. 2e). Compared to other β cells, Cluster 1 β cells were enriched in genes related to the ‘response to topologically incorrect protein’ and ‘response to ER stress’ pathways and exhibited downregulation of genes related to the ‘electron transport chain’, ‘cellular respiration’, ‘ATP metabolic process’, and ‘oxidative phosphorylation’ pathways (Fig. 4c). Cluster 2 β cells were enriched in genes related to the ‘negative regulation of cell population proliferation’ pathway and exhibited downregulation of genes related to the ‘transport vesicle’ and ‘secretory vesicle’ pathways (Fig. 4d). More specifically, ER stress genes (*Ddit3, Fkbp11*, and *Sdf2l1*) and other stress-related genes (*Nupr1, Atf5, Atf3, Hspa1a, Hspa1b*, and *Dnajb1*) were upregulated in β cells from multiparous mice, suggesting that cellular stress is increased in multiparous β cells (Fig. 4e, f). Cluster 5, enriched with virgin β cell, was upregulated in pathways related to oxidative phosphorylation and ATP metabolic process while ER-related pathways were downregulated (Supplementary Fig. 2f, g). In summary, our scRNA-seq analysis revealed that β cells from mice that had undergone multiple pregnancies have the following features: (1) increased cellular stress, including ER stress; (2) impaired cellular respiration and secretory functions; and (3) reduced proliferative capacity.

Cellular senescence is a state of cell cycle arrest that can be induced by repeated cell division and increased cellular stress^23,24^. Our scRNA-seq analysis suggested that repeated β cell division during multiple pregnancies and increased cellular stress are likely to impair the proliferative capacity of β cells in multiparous mice. Indeed, the telomere length was shortened (Fig. 4g), and the expression levels of the senescence markers *Cdkn2a* (p16) increased in islets from multiparous mice (Fig. 4h). These findings suggest that multiple pregnancies increase cellular stress in β cells and that this cellular stress induces aging features and senescence in β cells.

### Multiparous women have a higher risk of diabetes

**Fig. 5:**
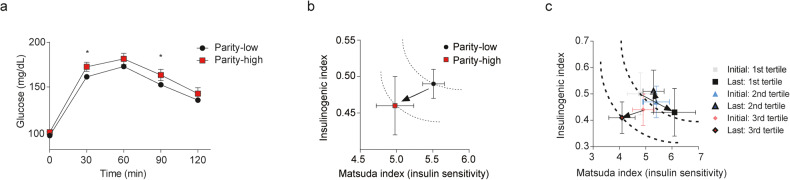
Women who have experienced multiple pregnancies have a higher risk of diabetes. Women who had one to three pregnancies were categorized as the parity-low group (*n* = 376), and those with four or more were categorized as the parity-high group (*n* = 79). **a** Results of the 75 g oral glucose tolerance test at 2 months postpartum. **b** Hyperbolic curve of the insulinogenic index and Matsuda index (insulin sensitivity) at 2 months postpartum. **c** Hyperbolic curve of parity-high women who were followed up for a median of 4.0 years after delivery (*n* = 77), classified into tertiles based on their change in body mass index from the initial follow-up (postpartum 2 months) to the last follow-up. (Data are expressed as the mean ± SEM. *P < 0.05, as determined by Student’s *t* test).

To examine the metabolic impact of multiparity on maternal metabolic physiology in humans, women with a history of gestational diabetes or gestational impaired glucose tolerance were examined with the 75 g oral glucose tolerance test at 2 months postpartum. Women who had one to three pregnancies were categorized as parity-low, and those who had four or more were categorized as parity-high. A total of 455 women were included in the analysis (parity-low, *n* = 376; parity-high, *n* = 79). Baseline characteristics, including glucose levels during gestation, exercise, lactation, and the duration of follow-up, were comparable between the two groups, but age and body mass index were higher in the parity-high group due to previous deliveries (Supplementary Table 1). At 2 months postpartum, parity-high women showed aggravated glucose profiles compared to parity-low women (Fig. 5a). The Matsuda index (insulin sensitivity) was ~10% lower in the parity-high group than in the parity-low group, and the insulinogenic index and disposition index (β cell function) tended to be lower in parity-high women, although these changes did not reach statistical significance (Fig. 5b, Supplementary Table 1). In a normal context in which β cells can physiologically compensate for insulin resistance, the insulinogenic index would increase when the Matsuda index decreases, resulting in no change in the disposition index. Similarly, we would expect the insulinogenic index to be higher in parity-high women since the Matsuda index was lower compared to the results obtained in parity-low women. However, the insulinogenic index was not higher in parity-high women, despite their decreased Matsuda index. Thus, the disposition index was lower in the parity-high women than in the parity-low women. These results suggest that β cells in parity-high women are unable to properly compensate for insulin resistance.

To further examine whether β cells of multiparous women can compensate adequately for insulin resistance, we investigated how changes in postpartum BMI affected metabolic profiles and β cell compensation for insulin resistance in multiparous women. Parity-high women were followed for a median of 4.0 years after the last delivery and classified into tertiles based on their change in body mass index (BMI) from the initial follow-up (postpartum 2 months) to the last follow-up. Intriguingly, the Matsuda index correlated well with the changes in BMI: The Matsuda index increased in the first tertile in which BMI decreased, decreased in the third tertile in which BMI increased, and was unaltered in the second tertile (Fig. 5c, Table 1). However, despite the decrease in the Matsuda index, the insulinogenic index did not increase in the third tertile. Therefore, the disposition index, which is a composite of the insulin secretory function of β cells considering the degree of insulin sensitivity, was lower in the third tertile than in the first and second tertiles. These findings support the human relevance of our findings that β cells of multiparous mice could not compensate for insulin resistance.

**Table 1:** Metabolic phenotypes of parity-high women categorized by their postpartum change in body mass index.

	Postpartum BMI change				(1st vs. 3rd)	(2nd vs. 3rd)	(1st vs. 2nd)
	1st tertile	2nd tertile	3rd tertile	Total	*P* value	*P* value	*P* value
*N*	27	24	26	77			
BMI (kg/m^2^)							
Initial	23.9 (0.5)	22.9 (0.5)	24.8 (0.8)	23.9 (0.4)	0.553	0.081	0.467
Last	23.1 (0.4)	22.7 (0.6)	25.9 (0.8)	23.9 (0.4)	0.004*	0.001*	0.876
Change	−0.9 (0.3)	−0.1 (0.2)	1.1 (0.3)	0.0 (0.2)	<0.001*	0.020*	0.129
AUC (Glucose)							
Initial	20148 (1449)	17988 (823)	18564 (592)	18940 (605)	0.524	0.922	0.319
Last	18821 (1548)	18986 (1319)	20735 (880)	19519 (740)	0.537	0.612	0.995
Change	−1327 (599)	999 (1013)	2172 (624)	579 (460)	0.004*	0.524	0.082
Matsuda index							
Initial	4.8 (0.5)	5.4 (0.5)	4.9 (0.4)	5.0 (0.3)	0.994	0.753	0.685
Last	6.1 (0.8)	5.3 (0.4)	4.1 (0.5)	5.2 (0.4)	0.044*	0.344	0.647
Change	1.2 (0.8)	−0.1 (0.5)	−0.9 (0.4)	0.1 (0.4)	0.045*	0.619	0.310
Insulinogenic index							
Initial	0.50 (0.08)	0.47 (0.06)	0.44 (0.06)	0.47 (0.04)	0.846	0.946	0.973
Last	0.43 (0.09)	0.51 (0.08)	0.41 (0.06)	0.45 (0.05)	0.982	0.685	0.788
Change	−0.07 (0.09)	0.04 (0.07)	−0.03 (0.08)	−0.02 (0.05)	0.998	0.832	0.870
Disposition index							
Initial	2.2 (0.4)	2.5 (0.4)	2.0 (0.3)	2.2 (0.2)	0.899	0.598	0.852
Last	2.4 (0.4)	2.7 (0.5)	1.4 (0.2)	2.2 (0.2)	0.033*	0.031*	0.796
Change	0.3 (0.5)	0.3 (0.5)	−0.6 (0.3)	0.0 (0.3)	0.270	0.336	0.990

Parity-high women were followed for a median of 4.0 years after delivery (*n* = 77) and classified into tertiles by the change in body mass index from the initial follow-up (postpartum 2 months) to the last follow-up. Metabolic phenotypes, including the area under the curve (AUC) for the oral glucose tolerance test and the insulinogenic index, disposition index (β cell function), and Matsuda index (insulin sensitivity), were compared. (Data are expressed as the mean ± SEM. *P < 0.05, as determined by ANOVA with Tukey’s post hoc test).

## Discussion

During pregnancy, women experience severe but physiological insulin resistance that can threaten their metabolic health after delivery. However, epidemiological studies have failed to agree on whether/how the risk of postpartum diabetes correlates with the number of pregnancies: Some found a positive correlation^3,4^, while others found a negative correlation^25,26^ or no association^27,28^. Moreover, the studies that reported a positive correlation between parity and the risk of diabetes failed to elucidate any detailed mechanism through which multiple pregnancies might lead to the deterioration of glucose tolerance.

In this study, we established a multiparity mouse model and longitudinally evaluated metabolic phenotypes. Mice that underwent multiple pregnancies developed insulin resistance over time, but their β cells were not able to compensate for the insulin resistance because of the impaired proliferative capacity. RNA-seq analyses revealed that multiple pregnancies induced cellular stress and aging features in β cells, which eventually resulted in β cell failure. Prospective human studies further supported our mouse findings by revealing that β cells failed to compensate for insulin resistance in multiparous women. Together, our findings show that multiparity increases the risk of diabetes in postpartum women by reducing the ability of their β cells to compensate for insulin resistance.

The age-related decline in β cell proliferation has been well described^29–35^. The expression of cyclin kinase inhibitor p16 (*Cdkn2a*) increases with aging; this prevents the entry of β cells into the cell cycle and promotes their maturation to enhance insulin secretion^31,35^. In a study from Helman et al., the forced induction of p16 and senescence in pancreatic β cells led to improved glucose-stimulated insulin secretion and increased expression of Gck^31^. In the present study, we show that insulin secretion was increased in islets from multiparous mice at 3 weeks after the last delivery and that this was accompanied by increased expression of p16 and *Mafa*. This finding suggests that during the initial phase of metabolic stress, β cell maturation and senescence occur concomitantly, followed by functional impairment upon further metabolic stress. In this context, although the metabolic phenotype of multiparous mice was not yet deteriorated at 3 weeks after the last delivery, the proliferative capacity of these β cells was already impaired at this time point. The multiparous β cells failed to compensate for the insulin resistance that developed afterward, as the multiparous mice gained weight. Furthermore, our RNA-seq analysis at 3 weeks after the last delivery revealed that cellular stress was already increased and cellular senescence was induced in β cells of multiparous mice before insulin resistance developed. Notably, insulin secretion was normal in islets from multiparous mice at 3 weeks after the last delivery; at 16 weeks after the last delivery, however, the basal insulin secretion of islets from multiparous mice under low-glucose conditions was elevated, as is often seen in cases of β cell aging or other β cell defects^31,34,36,37^. Taken together, the results from our animal study indicate that multiple pregnancies induce cellular senescence in β cells, and these β cells cannot compensate for insulin resistance, although they can maintain insulin secretory function for a while because they have already lost their proliferative capacity.

In this study, we conducted a comprehensive investigation into the alterations observed in the transcriptome landscape of islets in response to repeated delivery. Notably, we observed the upregulation of *Lrrc55* and *Matn2* in multipara islets, consistent with previous findings of their upregulation in pregnant islets^9,38^ or androgen receptor knockout islets^39^ (Supplementary Fig. 1b, d). *Lrrc55* has been implicated in the attenuation of ER stress-related pathways in β cells^38^, suggesting that its upregulation may be associated with an increased subpopulation of stressed β cells in multipara islets (Fig. 4). Furthermore, we observed upregulation of *Rerg* in S-961-injected multipara islets. *Rerg* has previously been shown to have an inhibitory role in cell proliferation in breast and nasopharyngeal cancer, particularly in an estrogen-dependent manner^40,41^. These genes hold promise as potential effector genes and may provide mechanistic insights into β cell replicative capacity. Further investigations into the functions of these genes are warranted.

In our single-cell RNA-seq analysis, we observed that multipara-enriched β cell clusters exhibited upregulation of genes associated with ‘response to ER stress’ and ‘negative regulation of cell population proliferation’ pathways, while they exhibited downregulation of genes related to ‘cellular respiration’ and ‘secretory vesicle’ pathways. These transcriptomic changes align with those observed in other models of β cell failure^42,43^. Interestingly, we observed upregulation of *Atf3* in multipara β cells, which is a gene known to induce cellular senescence through the regulation of epigenetic changes^44^. Furthermore, the *Atf4/p16* signaling pathway has been implicated in mediating ER stress-driven cellular senescence^45^. It is plausible that multipara β cells share common molecular pathways associated with ER stress-driven cellular senescence.

Cellular senescence is a state in which cells cease to divide and undergo a group of functional changes^23,46^. Metabolic stress, including chronological aging and insulin resistance, has been shown to induce cellular senescence in β cells, and researchers have attempted to use senolytic agents to improve metabolic phenotypes^47–49^. The expression of p16 increased with advanced age or the presence of diabetes mellitus in mouse and human pancreatic islets^35,47^. In human islet microarray data, subjects with diabetes had higher expression of Cdkn2a than those without diabetes (data not shown)^50^. Administration of a senolytic, ABT-263, specifically reduced p16 expression in islets and improved glucose tolerance and the genetic identity of β cells^47^. In terms of β cell proliferation, age-dependent declines in human β cell proliferation at the basal state^33^ and in adaptive β cell proliferation to partial pancreatectomy or low-dose streptozotocin in rodents have been well described^51^. Overexpression and genetic ablation of p16 result in the repression and induction of β cell proliferation, respectively^35^. These findings provide a potential link between β cell senescence from multiparity and an increased risk of postpartum diabetes and suggest the potential role of senolysis in recovering proliferative capacity. Further studies are required to explore the potential of senolytic therapies in rejuvenating the proliferative capacity of senescent β cells.

Epidemiologic studies have identified various risk factors for postpartum type 2 diabetes mellitus, including maternal age, prepregnancy body weight, and genetic predisposition, which are nonmodifiable factors^52,53^. On the other hand, modifiable risk factors such as postpartum weight change, lactation, physical activity levels, and dietary factors have been identified. Postpartum weight loss and lactation have shown benefits in improving insulin sensitivity and enhancing insulin secretory function^10,17^. Additionally, adhering to a healthier diet, such as the Mediterranean diet, has been associated with a reduced risk of developing postpartum diabetes^54^. The Diet, Exercise, and Breastfeeding Intervention (DEBI) study, an interventional trial, implemented a comprehensive approach to achieve postpartum weight goals through healthy eating, physical activity, and breastfeeding, resulting in improved glycemic control^54^. Postpartum weight loss was linked to improvements in fasting and 2 h glucose levels, as well as the homeostasis model assessment of insulin resistance. In our study, we observed that multipara women who experienced weight loss demonstrated improved insulin sensitivity. Conversely, women who gained weight during the study period exhibited an increase in insulin resistance, without a compensatory rise in insulin secretion. These findings suggest that weight loss in multipara women may positively impact their insulin sensitivity, potentially reducing the risk of developing insulin resistance-related conditions.

There are limitations to this study. First, we removed the pups from the cage to minimize the metabolic effects of lactation^10^. Although there was no difference in the activity (as shown in Fig. 1h), the separation of maternal mice from their pups may have caused psychological stress. Second, there are differences between mouse and human maternal physiology. Specifically, mice typically have multiple fetuses, approximately 10, whereas humans usually have a single fetus. Despite these limitations, we have presented human data showing that women with a higher number of pregnancies have higher glucose levels and impaired β cell function in those who gained weight after delivery. We believe that the metabolic phenotyping and bioinformatics analysis of rodent β cells provide valuable information for understanding maternal physiology.

In summary, we herein show that multiple pregnancies induce cellular stress and aging in β cells, rendering them unable to compensate for insulin resistance. Insulin resistance and adiposity increase after multiple pregnancies, but the loss of the proliferative capacity of β cells to compensate for insulin resistance is more critical for the development of glucose intolerance. Future studies on the modulation of β cell aging will provide new insights into maternal metabolism.

### Supplementary information

Supplementary Data

## Data Availability

All data that support the findings of this study are available from the authors on reasonable request. Bulk RNA-seq and single-cell RNA-seq data can be accessed via the Gene Expression Omnibus (GSE234740 and GSE 234741).
