# Author Correction: Multiparity increases the risk of diabetes by impairing the proliferative capacity of pancreatic β cells

**DOI:** 10.1038/s12276-023-01143-5

**Published:** 2023-12-19

**Authors:** Joon Ho Moon, Joonyub Lee, Kyun Hoo Kim, Hyun Jung Kim, Hyeongseok Kim, Hye-Na Cha, Jungsun Park, Hyeonkyu Lee, So-young Park, Hak Chul Jang, Hail Kim

**Affiliations:** 1 Graduate School of Medical Science and Engineering, Korea Advanced Institute of Science and Technology, Daejeon, 34141 Korea; 2 Department of Internal Medicine, Seoul National University Bundang Hospital, Seoul National University College of Medicine, Seoul, Korea; 3 Division of Endocrinology and Metabolism, Department of Internal Medicine, Seoul St. Mary’s Hospital, College of Medicine, The Catholic University of Korea, Seoul, Republic of Korea; 4 Biomedical Research Center, Korea Advanced Institute of Science and Technology, Daejeon, 34141 Korea; 5 Department of Biochemistry, College of Medicine, Chungnam National University, Daejeon, Korea; 6 Department of Physiology, College of Medicine, Yeongnam University, Daegu, Korea

**Keywords:** Gestational diabetes, Type 2 diabetes

Correction to: *Experimental & Molecular Medicine* 10.1038/s12276-023-01100-2 published online 31 October 2023

The wrong Supplementary file was originally published with this article; it has now been removed.

