# Supplementary material for: Multiparity increases the risk of diabetes by impairing the proliferative capacity of pancreatic β cells
Source: Exp Mol Med. 2023 Oct 31;55(10):2269–80. doi: 10.1038/s12276-023-01100-2 (PMC10618440; doi:10.1038/s12276-023-01100-2)
Supplement: Supplementary file 1 — Supplementary Data [file 12276_2023_1100_MOESM1_ESM.pdf]

Supplementary Methods

Animal experiments

C57BL/6J (The Jackson Laboratory) mice were used for the animal studies. The mice were housed and bred in a specific-pathogen-free facility under a 12-hour light and dark cycle with isothermal and isohumid conditions. A standard chow diet (Research Diets, Inc., D10001) and water were given *ad libitum*.

For the multiparity model experiments, 9-week-old female mice (C57BL/6J, The Jackson Laboratory) were mated and gave birth three times consecutively (multipara), and their virgin littermates were used as controls (virgin). To exclude the potential effects of lactation [1], pups were immediately removed from the cage at the day of delivery.

Glucose dynamics

For the intraperitoneal glucose tolerance test (GTT), mice were fasted overnight and D-glucose (2 g/kg) was administered intraperitoneally. Blood glucose was serially measured from the tail vein at 0, 15, 30, 60, 90, and 120 min. For the in vivo glucose-stimulated insulin secretion (GSIS) experiments, mice were fasted overnight and D-glucose (2 g/kg) was intraperitoneally injected. Blood was collected in heparinized tubes at 0 and 15 min from the tail vein. The collected blood was centrifuged for 10 min at 1,500g and 4°C, and the supernatant was collected. For the insulin tolerance test (ITT), mice were fasted for 6 hours and then injected with 0.75 U/kg of Humulin R (Lilly). Blood glucose was serially measured from the tail vein at 0, 15, 30, 45, 60, 75, and 90 min. The insulinogenic index was calculated from the in vivo GSIS test as: (insulin [15 min] − insulin [0 min])/(glucose [15 min] − glucose [0 min]). Blood glucose was measured with a glucometer (Allmedicus, AGM-3000) and plasma insulin was measured by enzyme-linked immunosorbent assay (ELISA; ALPCO, 80-INSMSU-E01).

Hyperinsulinemic-euglycemic clamp study

The hyperinsulinemic-euglycemic clamp study was performed as described previously [2]. The mice were pre-catheterized and non-anesthetized while the clamp study was performed. Somatostatin was infused at 6 ug/kg/min to suppress endogenous insulin secretion. Humulin R (Lilly) was infused at 15 pmol/kg/min and 20% glucose was infused to maintain the plasma glucose concentration at 6 mM. To measure whole-body glucose turnover, radiolabeled [3-3H] glucose (0.1 μCi/min, PerkinElmer) was infused. To measure the tissue glucose uptake, 2-deoxy-D-[1–14C] glucose (10 μCi, PerkinElmer) was injected. Hepatic glucose production (HGP) was calculated by subtracting the glucose infusion rate (GIR) from the whole-body glucose uptake rate. Plasma glucose levels were measured by a glucometer (Analox, GM9), and plasma insulin concentrations were measured by ELISA (Merck, EZRMI-13K).

Islet studies

Pancreatic islets were isolated as described previously [3]. For ex vivo GSIS, the retrieved islets were incubated in RPMI-1640 medium (Thermo Fisher Scientific, 11875101) mixed with 10% fetal bovine serum (Thermo Fisher Scientific, 16000044) and 100 U/ml penicillin-streptomycin (Thermo Fisher Scientific, 15070063) for 4 hours at 37°C/5% CO_2 in a humidified incubator. The islets were then incubated in 2.8 mM glucose Krebs-Ringer-HEPES (KRH) buffer for 30 minutes and transferred to a non-coated plate (12-well, 10 islets per mouse). The islets were incubated for 15 minutes in 2.8 mM glucose (basal, low) or 16.8 mM (stimulated, high) glucose-containing KRH buffer, and the secreted insulin level was measured. The islets were then sonicated and incubated in acid-ethanol (1.5% HCl in 100 ml of 70% ethanol) for 18 hours at 4°C for intracellular insulin extraction. The same volume of 1

M tris-Cl buffer (pH 8.0) was added for neutralization. All supernatants were immediately snap-frozen with liquid nitrogen and were kept frozen at −80°C until the ELISA experiments. Insulin secretion was normalized to the content of insulin extracted from the islets.

Quantitative reverse transcription-PCR (qRT-PCR)

One microgram of total RNA was applied to construct a cDNA library using a High-Capacity cDNA Reverse Transcription Kit (Applied Biosystems) following the provided instructions. The generated cDNA was mixed with Fast SYBR Green Master Mix (Applied Biosystems) and qRT-PCR was performed with a Viia 7 Real-time PCR System (Applied Biosystems).

RNA sequencing

The cDNA library sizes were measured using a DNA 1000 chip (Agilent Technologies 2100 Bioanalyzer). The prepared libraries were sequenced with a NovaSeq 6000 (Illumina), which generated 100-bp paired ends. The total reads per bases ranged from 7,100,000,000 to 7,900,000,000 bp, and the Q30 of all samples was above 94%.

Single-cell RNA sequencing

Islets were incubated for 30 minutes in RPMI-1640 medium supplemented with 10% fetal bovine serum and 100 U/ml penicillin-streptomycin at 37°C/5% CO2 in a humidified incubator, and then dissociated to the single-cell level with gentle pipetting in the presence of 0.25% Trypsin (Hyclone, SH30042.01) for 5 minutes. The cell viability and concentration of dissociated cells were evaluated using a Countess II Automated Cell Counter (AMQAX1000, Invitrogen). Samples with viability over 80% were concentrated to 800–1000 cells/μL. Dissociated cells were administered to the 10X Genomics Chromium system to generate

nanoliter-scale droplets containing uniquely barcoded beads called GEMs (Gel Bead-In EMulsions) and cDNA libraries were constructed using Chromium Single Cell 3' Reagent Kits (v3.1 Chemistry) following the manufacturer's instructions. The library size was analyzed with a DNA 1000 chip, and each library was sequenced using a HiSeq X Ten (Illumina). The Q30 was over 90% and the total read counts ranged from 384,000,000 to 406,000,000. The sequenced fastq data were aligned with Cell Ranger (3.1.0). The aligned data were analyzed by Seurat v3.0 after Gm42418 was removed from the gene-cell matrix (correlating genes were checked before the removal). Ambient RNAs were removed using SoupX (v1.4.5) [4]. Cells that met the criteria of nGene_RNA >200, nFeature_RNA <6000, nCount_RNA <60000, and percent.mt <10 were used for the analysis, and transcripts were log-normalized. Differential gene expression was evaluated using MAST (v.1.12.0) [5] and gene set enrichment analysis (GSEA) was performed with fgsea (v.1.12.0) [6].

Immunostaining

Mice were sacrificed and pancreatic tissues were harvested, fixed with 10% formalin for 4 hours at room temperature, and washed for 1 hour with deionized water at room temperature. The fixed pancreata were processed with an automatic tissue processor (Leica, TP1020), embedded in paraffin, whole-sectioned at 4-μm thickness, and mounted on glass slides. For liver and fat, tissues were formalin-fixed overnight and then washed for 1 hour in deionized water.

For immunofluorescence staining, slide-mounted samples were rehydrated and subjected to antigen retrieval in sodium citrate buffer [7]. Non-target epitopes were blocked with 2% donkey serum (The Jackson Laboratory) in PBS (phosphate-buffered saline) for 1 hour at room temperature. Anti-insulin (guinea pig, Dako, 1:500) or anti-Ki-67 (rabbit, Abcam, 1:1000) were applied as primary antibodies overnight at 4°C. The slides were washed

with PBS, and secondary antibodies [Alexa 488-conjugated donkey anti-mouse IgG (Jackson ImmunoResearch, 1:500), Alexa 594-conjugated donkey anti-rabbit IgG (Jackson ImmunoResearch, 1:500)] were applied for 2 hours under a light shield at room temperature. The slides were washed with PBS and stained with DAPI (Invitrogen, 1:1000) for 5 minutes, and cover glasses were applied with fluorescence mounting medium (Dako).

For immunohistochemical staining, pancreas sections were antigen-retrieved as described for immunofluorescence staining and washed in PBS. Endogenous peroxidases were blocked with BLOXALL (Vector, SP-6000-100) for 10 minutes at room temperature. The slides were blocked with 2% goat serum, incubated with anti-insulin (guinea pig, Dako, 1:1000) overnight at 4°C, and washed with PBS. Immunohistochemistry was performed using a VECTASTAIN ABC kit (Vector Laboratories, PK-6100) and DAB (Vector Laboratories, SK-4100) following the manufacturer's protocols, and the slides were counterstained with hematoxylin and eosin.

Measurement of β cell mass and proliferation

For β cell mass measurement, pancreas sections were collected every 80 μm and immunohistochemical staining was performed for insulin. Images were acquired with a JuLI Stage recorder (NanoEnTek). The insulin immuno-reactive and whole pancreatic areas were measured, and the former divided by the latter was calculated to assess β cell mass. For β cell proliferation, Ki-67 and insulin were immunostained in pancreas sections obtained every 80 μm. The percentage of Ki-67 and insulin co-positive cells over the percentage of insulin-positive cells was calculated as the β cell proliferation rate.

Human studies

All pregnant women were screened for GDM with a 50-g oral glucose challenge test between 24 and 28 weeks of gestation; a positive screen was defined as a 1-hour glucose

value ≥130 mg/dL. Women with a positive screen were given a 3-hour 100 g oral glucose tolerance test (OGTT) for diagnosis of GDM. The diagnoses of GDM and GIGT were made using the recommendations of the Third International Workshop-Conference on Gestational Diabetes Mellitus [8]. Two or more of the following criteria for GDM and one of the following criteria for GIGT were required for the diagnosis: fasting plasma glucose ≥105 mg/dL, 1-hour glucose ≥190 mg/dL, 2-hour glucose ≥165 mg/dL, or 3-hour glucose ≥145 mg/dL.

Women with GDM or GIGT who visited the initial postpartum evaluation were enrolled in this study. The initial postpartum follow-up visit was performed at 2 months postpartum, and annual follow-up visits were made thereafter. Subjects who had persistent diabetes at the initial visit were excluded. Among the eligible subjects, women who had one to three pregnancies were categorized as 'parity-low' and those with four or more were categorized as 'parity-high'. A total of 455 women were included in the analysis (parity-low, n = 376; parity-high, n = 79), having completed more than one follow-up examination and undergone the OGTT. The median duration of follow-up was 4.0 (interquartile range, 2.2 – 5.0) years after delivery. Parity-high women with missing data after the initial postpartum follow-up (n=2) were excluded from further analysis.

Postpartum follow-up examinations

Face-to-face interviews were conducted at the first postpartum examination using a standardized questionnaire that included past medical and reproductive history. Obstetric history, including parity and pregestational weight, was retrieved from the patient's medical records. A standard 75 g OGTT was performed at each postpartum follow-up visit. Anthropometric measures, including body weight and height, were measured at each visit.

Metabolic assessment

A metabolic assessment, including a 75-g OGTT, was performed after an overnight fast. The plasma glucose concentrations were enzymatically measured with an automated analyzer (Yellow Springs Instrument Co.), using the glucose-oxidase method. The plasma insulin concentrations were measured by a radioimmunoassay (Linco Research, Inc.). The insulin sensitivity was assessed by the Matsuda index as follows: 10,000 / √[(fasting glucose) × (fasting insulin) × (mean glucose) × (mean insulin)] [9]. The insulinogenic index was used to estimate insulin secretion as follows: (insulin [30 min] - insulin [0 min]) / (glucose [30 min] - glucose [0 min]) [10]. The disposition index was used to evaluate the composite of insulin secretion considering the degree of insulin sensitivity as follows: (Matsuda index) × (insulinogenic index) [11].

Supplementary Figures

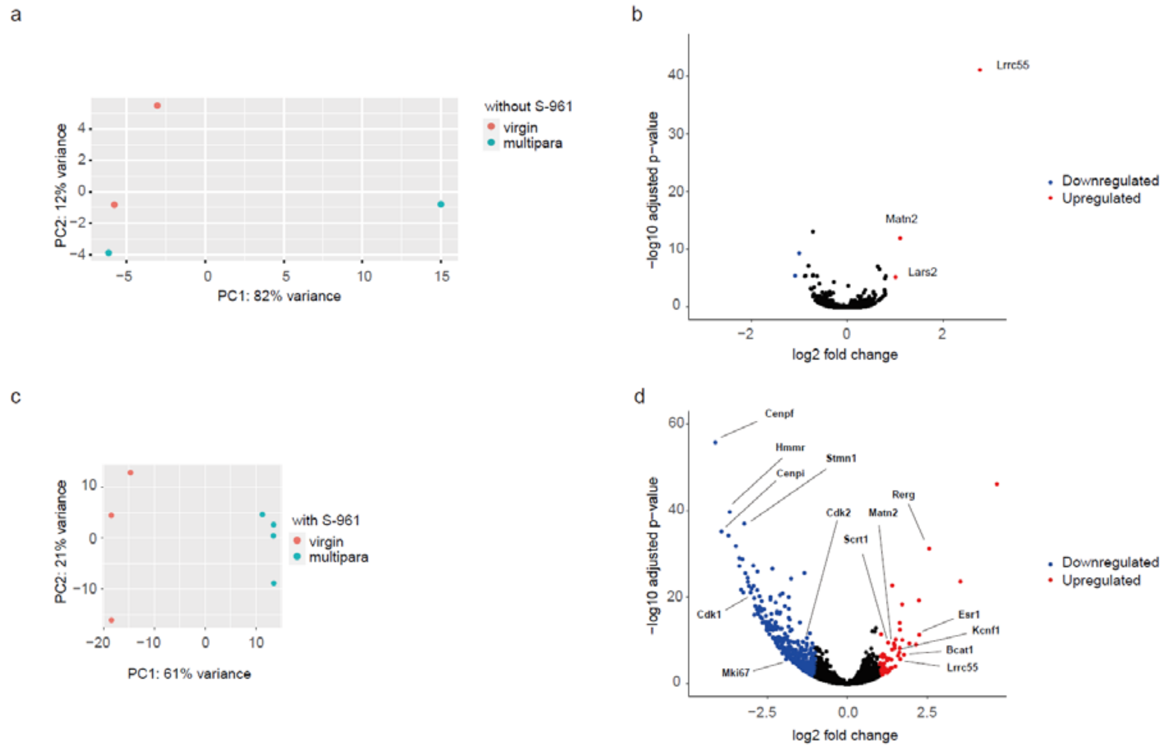

Supplementary Fig 1. Bulk RNA-seq analysis in islets from virgin and multiparous mice

(a, b) Bulk RNA-seq analysis of islets from virgin and multiparous mice (non-S-961-injected, n=2 per group). (c, d) Bulk RNA-seq analysis of islets from virgin and multiparous mice (S-961-injected, n≥3 per group). (a, c) PCA plots in which virgin (red) and multiparous (green) samples are represented as dots. (b, d) Differentially expressed genes are presented as a volcano plot (blue: downregulated, red: upregulated, black: non-significant, cut off: log2 fold change > 1 or < -1 with adjusted p-value < 0.05).

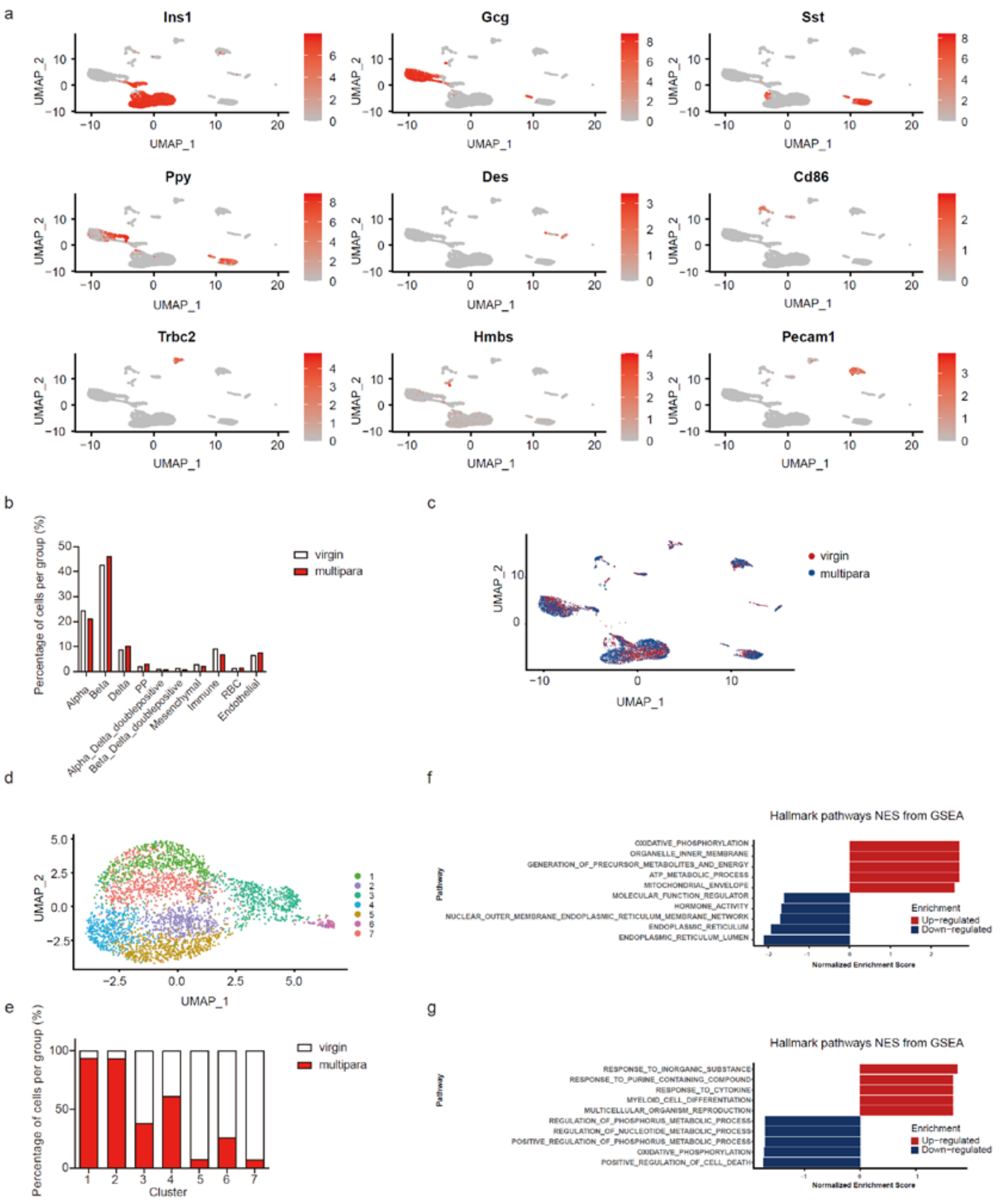

Supplementary Fig 2. Single-cell RNA-seq analysis in islets from virgin and multiparous mice

(a-g) Single-cell RNA sequencing of islets from virgin and multiparous mice (n≥2 per group). (a) Representative marker genes were screened for each cell type in islets: Ins1 for β cells, Gcg for α cells, Sst for δ cells, Ppy for PP cells, Des for mesenchymal cells, Cd86, Trbc2 for immune cells, Hmbs for red blood cells, and Pecam1 for endothelial cells. (b) A between-group comparison of the proportion of each cell type. (c) Expression patterns of islet cells from virgin and multiparous mice plotted in a UMAP. (d) An unsupervised clustering of β cells from virgin and multiparous mice was performed based on their expression patterns. (e) A between-group comparison of the proportion of each β cell cluster. (f, g) Pathways of genes enriched in (f) Cluster 5 and (g) Cluster 7 were analyzed using gene set enrichment analysis.

Supplementary Tables

	Parity-low (n=376)	Parity-high (n=79)	P-value
**Anthropomety**			
**Age at delivery (years)**	30.9 (0.2)	33.1 (0.5)	<0.001*
**Body mass index, pre-gestational (kg/m^2)**	22.3 (0.2)	23.3 (0.4)	0.015*
**Parity (n)**	1.7 (0.1)	5.0 (0.2)	<0.001*
**Duration of follow-up (days)**	1349 (36)	1440 (83)	0.300
**Exercise (%)**	227 (60.4%)	50 (63.3%)	0.629
**Lactation (%)**	176 (46.8%)	41 (53.2%)	0.304
**Gestational weight gain (kg)**	11.7 (0.3)	10.6 (0.6)	0.162
**Glycemic profile during pregnancy**			
**1-hour 50-g glucose challenge (mg/dL)**	165 (1.5)	171.8 (5.2)	0.210
**3-hour 100-g glucose tolerance test (mg/dL)**			
**Glucose (fasting)**	91.8 (1.0)	94.8 (2.3)	0.221
**Glucose (1 hour)**	188.9 (1.9)	193.1 (4.6)	0.221
**Glucose (2 hour)**	176.9 (1.9)	180.1 (4.2)	0.488
**Glucose (3 hour)**	152.1 (1.9)	149.9 (5.1)	0.642
**Insulin (fasting)**	39.3 (2.4)	37.6 (5.1)	0.774
**Insulin (1 hour)**	95.5 (3.5)	98.0 (8.9)	0.781
**Insulin (2 hour)**	110.8 (3.7)	116.7 (9.2)	0.524
**Insulin (3 hour)**	99.4 (3.3)	103.8 (8.5)	0.601
**Glycemic profile at 2 months postpartum**			
**Matsuda index (insulin sensitivity)**	5.5 (0.2)	5.0 (0.3)	0.085
**Insulinogenic index**	0.49 (0.02)	0.46 (0.04)	0.576
**Disposition index**	2.6 (0.2)	2.2 (0.2)	0.126

Supplementary Table 1. Clinical characteristics of the human cohort

Women who had one to three pregnancies were categorized as parity-low (n=376) and those with four or more were categorized as parity-high (n=79). Comparison of their baseline characteristics, including anthropometric information and glycemic profile data obtained from the 50-g glucose challenge and 100-g oral glucose tolerance test during pregnancy. Clinical characteristics including insulinogenic index, disposition index (β cell function), and Matsuda index (insulin sensitivity) at 2 months postpartum were compared. (Data are expressed as mean ± SEMs. *$P < 0.05$.)

Gene	Species		Primer sequence
**Cdkn2a**	Mouse	Forward	GCTTCTCACCTCGCTTGTCA
		Reverse	CCATCATCATCACCTGGTCC
**Mafa**	Mouse	Forward	CTTCAGCAAGGAGGAGGTCATC
		Reverse	GCGTAGCCGCGGTTCTT
**Ccnd1**	Mouse	Forward	GTGCTGCGAGCCATGCTCAA
		Reverse	TTCATGGCCAGCGGGAAGA
**Telomere**	Mouse	Forward	CGGTTTGTTTGGGTTTGGGTTTGGGTTTGGGTTTGGGTT
		Reverse	GGCTTGCCTTACCCTTACCCTTACCCTTACCCTTACCCT

Supplementary Table 2. Sequences of the primers used for qRT-PCR

Reference

1. Moon, J. H. *et al.* Lactation improves pancreatic beta cell mass and function through serotonin production. *Sci Transl Med* **12** (2020). https://doi.org/10.1126/scitranslmed.aay0455
2. Cha, H. N., Park, S., Dan, Y., Kim, J. R. & Park, S. Y. Peroxiredoxin2 Deficiency Aggravates Aging-Induced Insulin Resistance and Declines Muscle Strength. *J Gerontol A Biol Sci Med Sci* **74**, 147-154 (2019). https://doi.org/10.1093/gerona/gly113
3. Szot, G. L., Koudria, P. & Bluestone, J. A. Murine pancreatic islet isolation. *J Vis Exp*, 255 (2007). https://doi.org/10.3791/255
4. Satija, R., Farrell, J. A., Gennert, D., Schier, A. F. & Regev, A. Spatial reconstruction of single-cell gene expression data. *Nat Biotechnol* **33**, 495-502 (2015). https://doi.org/10.1038/nbt.3192
5. Finak, G. *et al.* MAST: a flexible statistical framework for assessing transcriptional changes and characterizing heterogeneity in single-cell RNA sequencing data. *Genome Biology* **16**, 278 (2015). https://doi.org/10.1186/s13059-015-0844-5
6. Sergushichev, A. A. An algorithm for fast preranked gene set enrichment analysis using cumulative statistic calculation. *bioRxiv*, 060012 (2016). https://doi.org/10.1101/060012
7. Kim, H. *et al.* PRMT1 Is Required for the Maintenance of Mature β-Cell Identity. *Diabetes* **69**, 355-368 (2020). https://doi.org/10.2337/db19-0685
8. Metzger, B. E. Summary and recommendations of the third international workshop-conference on gestational diabetes mellitus. *Diabetes* **40**, 197-201 (1991).
9. Matsuda, M. & DeFronzo, R. A. Insulin sensitivity indices obtained from oral glucose tolerance testing: comparison with the euglycemic insulin clamp. *Diabetes Care* **22**, 1462-1470 (1999).
10. Tura, A., Kautzky-Willer, A. & Pacini, G. Insulinogenic indices from insulin and C-peptide: comparison of beta-cell function from OGTT and IVGTT. *Diabetes Res Clin Pract* **72**, 298-301 (2006). https://doi.org/10.1016/j.diabres.2005.10.005
11. Retnakaran, R., Qi, Y., Goran, M. I. & Hamilton, J. K. Evaluation of proposed oral disposition index measures in relation to the actual disposition index. *Diabetic medicine : a journal of the British Diabetic Association* **26**, 1198-1203 (2009). https://doi.org/10.1111/j.1464-5491.2009.02841.x
